# Retinoic acid is dispensable for meiotic initiation but required for spermiogenesis in the mammalian testis

**DOI:** 10.1242/dev.201638

**Published:** 2023-07-13

**Authors:** Oleksandr Kirsanov, Taylor A. Johnson, Bryan A. Niedenberger, Taylor N. Malachowski, Benjamin J. Hale, Qing Chen, Brad Lackford, Jiajia Wang, Anukriti Singh, Karen Schindler, Brian P. Hermann, Guang Hu, Christopher B. Geyer

**Affiliations:** ^1^ Department of Anatomy and Cell Biology, Brody School of Medicine, East Carolina University, Greenville, NC 27858, USA; ^2^ Epigenetics and Stem Cell Laboratory, National Institute of Environmental Health Sciences, Durham, NC 27709, USA; ^3^ Department of Neuroscience, Developmental and Regenerative Biology, The University of Texas at San Antonio, San Antonio, TX 78249, USA; ^4^ Department of Genetics, Rutgers, The State University of New Jersey, Piscataway, NJ 08854, USA; ^5^ East Carolina Diabetes and Obesity Institute at East Carolina University, Greenville, NC 27834, USA

**Keywords:** Spermatogenesis, Retinoic acid, Meiosis, Testis

## Abstract

Retinoic acid (RA) is the proposed mammalian ‘meiosis inducing substance’. However, evidence for this role comes from studies in the fetal ovary, where germ cell differentiation and meiotic initiation are temporally inseparable. In the postnatal testis, these events are separated by more than 1 week. Exploiting this difference, we discovered that, although RA is required for spermatogonial differentiation, it is dispensable for the subsequent initiation, progression and completion of meiosis. Indeed, in the absence of RA, the meiotic transcriptome program in both differentiating spermatogonia and spermatocytes entering meiosis was largely unaffected. Instead, transcripts encoding factors required during spermiogenesis were aberrant during preleptonema, and the subsequent spermatid morphogenesis program was disrupted such that no sperm were produced. Taken together, these data reveal a RA-independent model for male meiotic initiation.

## Teaser

**Summary:** *In vitro* and *in vivo* models paired with loss- and gain-of-function approaches reveal that after triggering the spermatogonial differentiation program, retinoic acid is dispensable for meiotic initiation in the mammalian testis.

## INTRODUCTION

Meiosis is essential for sexual reproduction, and its specialized processes have been intensely studied in both lower eukaryotes and mammals. In mammals, two landmark studies using fetal mice identified retinoic acid (RA) as the long sought-after ‘meiosis-inducing factor’ (MIS) (Bowles et al., 2006; Koubova et al., 2006). However, the relevance of these observations to male meiotic initiation in the postnatal testis is questionable. This is because in the postnatal mouse testis, a pulse of RA is essential for differentiation of mitotic spermatogonia (Haneji et al., 1983; van Pelt and de Rooij, 1991), which precedes meiotic initiation by 8.6 days (Oakberg, 1956b). Therefore, the conclusion that a second pulse of RA is the sole molecular trigger for meiotic initiation is not supported by direct experimentation. Currently, the only evidence that RA directs male meiotic initiation is correlative activation of meiosis genes such as *Stra8* and *Rec8* (Koubova et al., 2014; Oulad-Abdelghani et al., 1996), which are themselves not required for meiotic initiation, but rather for meiotic progression (Xu et al., 2005; Anderson et al., 2008; Mark et al., 2008). Indeed, in *Stra8* KO mice on an inbred C57BL/6 background, preleptotene spermatocytes completed meiotic DNA replication but failed to load the meiotic cohesin REC8 (Anderson et al., 2008), whereas on a mixed genetic background preleptotene spermatocytes were formed, and they completed meiotic S-phase (Baltus et al., 2006; Anderson et al., 2008) and even reached pachynema (Mark et al., 2008). Therefore, the requirement for RA in meiotic initiation and progression needs to be critically examined using additional strategies.

Here, we used both *in vitro* and *in vivo* models paired with loss- and gain-of-function approaches to examine the requirement for RA as the instructive signal for male meiotic initiation. We discovered that, in the absence of RA, male germ cells initiated, progressed through and completed meiosis to form haploid spermatids. The initiation of meiosis in RA-deficient male germ cells was accompanied by rather modest changes in overall gene expression, and the activation of meiosis-related genes was largely unaffected. However, there were significant changes in expression of genes encoding factors involved in and required for the morphogenetic changes during the later developmental program of spermiogenesis that creates sperm. An examination of this process revealed spermatids in RA-deficient testes failed to properly elongate and, as a consequence, no sperm were produced. In conclusion, our data support an RA-independent model of meiotic initiation in the mammalian testis.

## RESULTS

### Synchronizing spermatogenesis to examine gene expression during spermatogonia differentiation and meiotic initiation

**Fig. 1.**
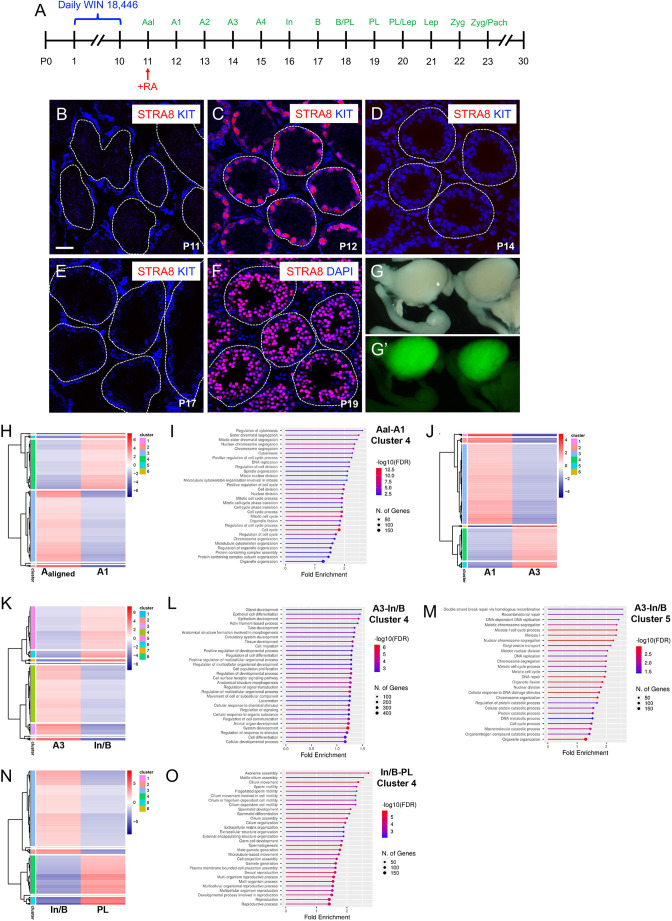
**Adapting a model of synchronized spermatogenesis to study spermatogonial differentiation and meiotic initiation *in vivo*.** (A) Synchronization timeline for treatments to generate RA-sufficient mice. (B-F) Testis sections from mice with RA-sufficient spermatogenesis are shown, with ages indicated on each image. Sections were immunostained for the indicated germ cell fate markers (colors indicated on each image) and nuclei were counterstained with DAPI (blue). Tubules are outlined. (G,G′) Testes from P60 and older Uchl1-eGfp transgenic mice are shown, illuminated by white (G) or fluorescent (G′) light. (H,J,K,N) Heatmaps showing log2Fold-change values of mRNA abundance difference between the noted cell populations (listed below). Fold-change is indicated by the color scale and unsupervised gene clusters are shown on the left. (I,L,M,O) Lollipop plots indicate Gene Ontology Enrichment Analysis of genes from noted clusters. Scale bar: 40 µm. Each experiment was repeated thrice and *n*≥4 mice were used for each experiment.

Currently, little is known about the requisite program of differentiation that spermatogonia must complete before meiotic initiation. To study this, we adapted an *in vivo* model of synchronized steady state spermatogenesis (Hogarth et al., 2013; Romer et al., 2018). As shown in the timeline (Fig. 1A), synchronization was accomplished by dosing mice with the potent and highly selective RA synthesis inhibitor WIN 18,446 beginning at postnatal day (P)1, before endogenous RA signaling and when testes contain only prospermatogonia – the precursors of spermatogonia (Culty, 2013; McCarrey, 2013). Daily WIN 18,446 treatment through P10 blocked normal spermatogonia differentiation, and thus testes at P11 were filled with STRA8^−^/KIT^−^ undifferentiated stem and progenitor spermatogonia (Fig. 1B). At P11, a single injection of exogenous RA induced all spermatogonia to differentiate (except spermatogonial stem cells, or SSCs, which are RA insensitive; Velte et al., 2019). RA is extremely labile, with a half-life in mice of less than 0.5 h (McPhillips et al., 1987; Nau, 1986), and therefore is not expected to persist in testes after P12. Twenty-four hours later in these ‘RA-sufficient’ testes, nearly all germ cells were STRA8^+^/KIT^+^ A_1_ differentiating spermatogonia (Fig. 1C) that proceeded through remaining stages [A_2-4_, intermediate spermatogonia (In) and type B spermatogonia (B)] of differentiation (Fig. 1D,E) and entered meiosis at P19 as STRA8^+^ preleptotene spermatocytes (Fig. 1F). These DMRT1^−^/HIF6^+^ spermatocytes completed meiosis to form round spermatids by P30 (Fig. S1A-C), elongating spermatids by P37 (Fig. S1F) and condensing spermatids by P43, which were clustered around lumina in preparation for spermiation (Fig. S1G).

Using this model system, we next identified gene expression changes at the transcriptome level before differentiation, at the onset, midpoint and end of differentiation, and at meiotic initiation. To isolate each distinct cell population for bulk RNA-seq, we used transgenic mice expressing EGFP downstream of the promoter for ‘ubiquitin carboxyl-terminal hydrolase L1’ (*Uchl1*) (Yasvoina et al., 2013). Created to label motor neurons, testes of these mice were also EGFP^+^ (Fig. 1G-G′), with consistent high levels in spermatogonia and low levels in preleptotene spermatocytes entering meiosis (Kirsanov et al., 2022). This consistent difference in fluorescence intensity enables isolation, from testes with synchronized spermatogenesis, of highly enriched populations of millions of EGFP^bright^ premeiotic spermatogonia and EGFP^dim^ preleptotene spermatocytes entering meiosis. As reported previously, FACS cell populations were 91-94% pure, which was verified by flow cytometry purity analysis (for EGFP) as well as by staining sorted cell aliquots with germ cell fate markers (Kirsanov et al., 2022). EGFP^bright^ In/B differentiating spermatogonia and EGFP^dim^ preleptotene spermatocytes from testes of *Uchl1-*EGFP mice with synchronized spermatogenesis were used for bulk RNA-seq to measure changes in mRNA abundance during spermatogonial differentiation and entry into meiosis. Four pairwise comparisons were made between temporally adjacent cell types, including undifferentiated spermatogonia (A_undiff_, or A_al_) versus A_1_ differentiating spermatogonia, A_1_ versus A_3_ differentiating spermatogonia, A_3_ versus In/B differentiating spermatogonia, and In/B versus preleptotene spermatocytes to identify differentially expressed genes (DEGs; log2 fold-change>1, and *P*_Adj_≤0.01; Table S1)*.* We found 1859 DEGs between A_undiff_ and A_1_ spermatogonia (1082 higher in A_undiff_, 777 higher in A_1_) (Fig. 1H). Messages encoding prototypical markers of stem and progenitor spermatogonia (e.g. *Id4*, *Nanos2*, *Tcl1*, *Upp1* and *Zbtb16*) declined in differentiating spermatogonia, whereas those encoding markers of differentiating spermatogonia (e.g. *Kit*, *Sohlh2* and *Stra8*) increased (Fig. 1H,I, Table S1). Notably, genes in cluster 4, which had elevated mRNA levels at the onset of differentiation, were significantly enriched for cell proliferation biological processes (e.g. regulation of cytokinesis, positive regulation of cell cycle process and regulation of cell division; Fig. 1J, Table S2). A relatively small number of DEGs were identified between A_1_ and A_3_ differentiating spermatogonia (554 DEGs, 383 higher in A_1_ and 171 higher in A_3_), which clustered into six groups based on expression pattern (Table S3). Surprisingly, GO analysis identified no significant enrichment of biological processes among genes in any of the six A_1_ versus A_3_ clusters, suggesting no significant biological changes are under way at the midpoint of spermatogonial differentiation. As spermatogonial differentiation progressed beyond the mid A_3_ into the late In/B stages, however, we observed more changes in mRNA levels (2461 DEGs, 1407 higher in A_3_, 1054 higher in In/B) that grouped into eight DEG clusters (Fig. 1K, Table S4). Pathway analysis of these clusters demonstrated mRNAs from genes involved in developmental programs were reduced in the A_3_-to-In/B transition (Fig. 1L, Table S4), whereas those involved in meiosis were increased at the In/B spermatogonia stage (Fig. 1M, Table S4). These data confirm activation of genes constituting the meiotic program during spermatogonial differentiation before meiotic entry. Immediately before meiotic entry, we observed the highest numbers of mRNA abundance changes between In/B spermatogonia and preleptotene spermatocytes (3349 DEGs, 2133 higher in In/B, 1216 higher in PL, Table S1), which grouped into six DEG clusters (Fig. 1N, Table S5). Biological pathways corresponding to translation control and a variety of biosynthetic pathways were over-represented among genes whose mRNA levels decreased upon meiotic initiation, whereas there was increased mRNA abundance for genes involved in spermiogenesis and sperm function (Fig. 1O, Table S5).

### RA-deficient spermatogonia both entered and completed meiosis *in vivo*

**Fig. 2.**
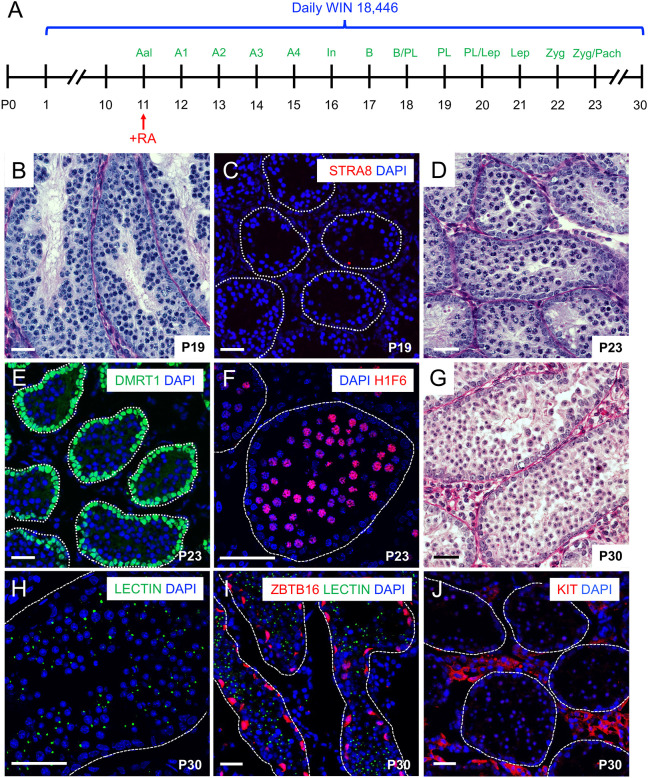
**Male germ cells initiate and complete meiosis in the absence of RA *in vivo*.** (A) Synchronization timeline for a single injection of 10 µl of exogenous RA (10 µg/µl) in DMSO (to induce differentiation) and continuous daily treatment with 100 µg/g WIN 18,446 to generate RA-deficient testes. (B-J) Testis sections from mice with RA-deficient spermatogenesis are shown, with ages indicated on each image. Sections in B, D and G were stained with periodic acid-Schiff. In C,E,F,H-J, sections were immunostained for the indicated germ cell fate markers (colors indicated on each image) or with lectin to mark spermatid acrosomes (in H,I); nuclei were counterstained with DAPI (blue). Tubules are outlined. Scale bars: 40 µm. Each experiment was repeated thrice and *n*≥4 mice were used for each experiment.

These transcriptome data revealed that: (1) activation of the gene expression program for many meiotic genes occurred during spermatogonial differentiation well before meiotic initiation; and (2) levels of many meiotic mRNAs were not significantly altered during preleptonema, when the second round of RA signaling presumably occurs (Endo et al., 2017; Hogarth et al., 2014). As the requirement for RA in male meiotic initiation has not been definitively tested, we modified the synchronization protocol into a loss-of-function approach to generate ‘RA-deficient’ testes by continuously blocking RA synthesis with WIN 18,446 after inducing differentiation at P11 (Fig. 2A). Loss of RA signaling *in vivo* at the time of meiotic initiation was confirmed in these testes using multiple approaches. First, RA-induced mRNAs (e.g. *Stra8* and *Rec8*; Koubova et al., 2014; Oulad-Abdelghani et al., 1996; Zhou et al., 2008) were not upregulated in preleptotene spermatocytes from WIN 18,446-treated mice, whereas those for the RA-independent meiotic strand exchange factor *Dmc1* were significantly elevated (Fig. S2A). Second, STRA8 protein was also undetectable, whereas SYCP3 was elevated in both RA-deficient and -sufficient testes (Fig. S2B). Third, morphologically normal STRA8^−^ preleptotene spermatocytes were formed in RA-deficient testes at P19 (Fig. 2B,C), on the same day as in control ‘RA-sufficient’ testes (Fig. 1F). Fourth, there were no KIT^+^ spermatogonia (indicating absence of RA-induced differentiation) from P19 to P23 (Fig. S3A-E). The expression of meiotic genes such as *Dmc1* and *Sycp3*, and formation of preleptotene spermatocytes in RA-deficient testes suggested onset of meiotic initiation was not reliant upon the signal provided by RA.

We next assessed meiotic progression in RA-deficient testes and found morphologically normal DMRT1^−^/H1F6^+^ zygotene/pachytene spermatocytes appeared by P23 (Fig. 2D-F) and round spermatids appeared on P30 (Fig. 2G-J), as seen in RA-sufficient testes (Fig. S1A-E). In stark contrast to control RA-sufficient testes that contained the next generation of KIT^+^ differentiating spermatogonia (Fig. S4), RA-deficient testes at P30 contained only two discrete populations of germ cells – lectin^+^ round spermatids and undifferentiated ZBTB16^+^/KIT^−^ spermatogonia (Fig. 2I,J). Importantly, spermatocytes from any potential subsequent waves of differentiation were never observed. These results provide further evidence that continuous WIN 18,446 treatment effectively blocked RA synthesis and thus further spermatogonial differentiation *in vivo*.

We next determined whether spermatogenic cells that apparently entered and completed meiosis in RA-deficient testes *in vivo* faithfully completed landmark cytological processes of meiosis. We first used flow cytometry to examine changes in DNA content. Single cell suspensions were generated from RA-sufficient and -deficient testes at P23 and P30, and an aliquot was incubated with Trypan Blue, which is excluded from live cells (Strober, 2015). Counts were performed using a hemocytometer and simply recorded and calculated. Cells were 89-92% viable.

**Fig. 3.**
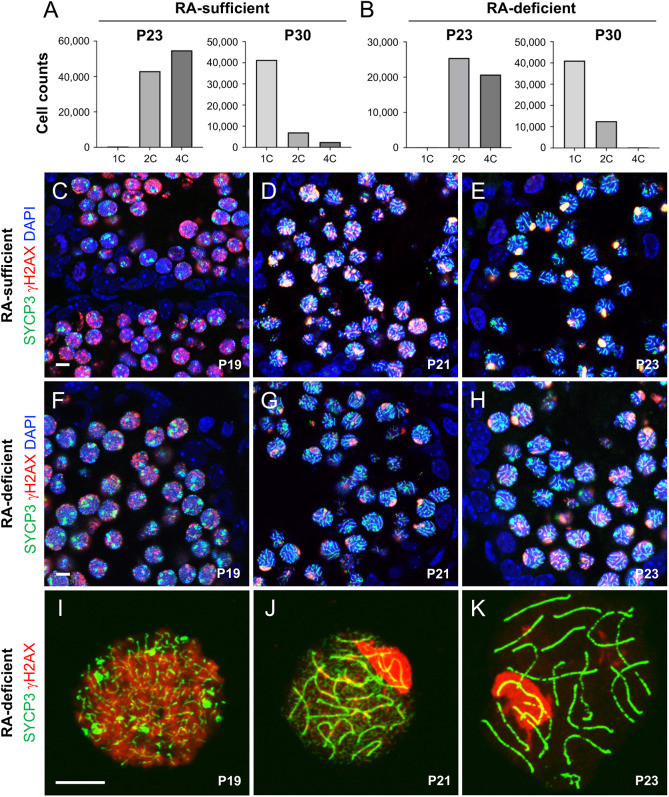
**Landmark cytological events of meiosis are faithfully completed in the absence of RA *in vivo*.** (A,B) DNA content was assessed by flow cytometry in RA-sufficient (A) and RA-deficient (B) testes from mice at P23 and P30. At P23, testes from both groups contained 2C and abundant 4C cells, indicating spermatocytes had indeed replicated their DNA in the absence of RA. At P30, testes from both groups contained abundant 1C and 2C cells, indicating haploid spermatids were indeed formed in the absence of RA. In addition, RA-sufficient P30 testes contained the next generation of 4C spermatocytes, whereas those from RA-deficient mice did not. (C-H) Testes containing spermatocytes from RA-sufficient (C-E) and RA-deficient (F-H) mice at the ages indicated on each image exhibited similarly normal γH2AX (red) and SYCP3 (green) localization patterns, and DAPI-labeled nuclei (in blue). (I-K) Meiotic chromosome spreads from RA-deficient testes had normal immunostaining patterns for γH2AX (red) and SYCP3 (green). Scale bars: 10 µm. Each experiment was repeated thrice and *n*≥4 mice were used for each experiment.

During preleptonema, 2N/2C spermatocytes replicate their DNA, becoming 2N/4C. At the end of meiosis I, 2N/4C diplotene spermatocytes divide twice, successively forming two 2N/2C secondary spermatocytes and four 1N/1C round spermatids. As expected, RA-sufficient testes from P23 mice contained 2C and 4C cells (somatic cells/spermatogonia and zygotene/pachytene spermatocytes, respectively), whereas those from P30 mice contained 1C, 2C and 4C cells (round spermatids, somatic cells+spermatogonia and spermatocytes from subsequent waves of differentiation, respectively) (Fig. 3A, Fig. S5). Testes from RA-deficient mice at P23 also contained 2C and 4C cells (Fig. 3B, Fig. S5), revealing zygotene/pachytene spermatocytes indeed replicated their DNA. By P30, testes from RA-deficient mice contained 1C and 2C cells, revealing the initial synchronized cohort of germ cells completed meiosis as haploid (1C) round spermatids (Fig. 3B, Fig. S5) in the absence of RA. Importantly, in contrast to RA-sufficient testes, P30 RA-deficient testes did not contain 4C spermatocytes, further confirming inhibition of RA signaling in these mice (Fig. 3B, Fig. S5). We next assessed whether spermatocytes in RA-deficient testes successfully completed key events during leptonema, zygonema and pachynema of the first meiotic prophase. Immunostaining for established markers of chromosome synapsis (SYCP3) and double-stranded break formation (γH2AX) revealed that spermatocytes in RA-sufficient testes (Fig. 3C-E) resembled those produced in RA-deficient testes (Fig. 3F-H). Likewise, meiotic chromosome spreads from RA-deficient testes from P19 to P23 demonstrated normal-appearing SYCP3^+^ synaptonemal complexes after P20, and γH2AX^+^ double-stranded breaks and XY, or sex body, localization as early as P21 (Fig. 3I-K).

**Fig. 4.**
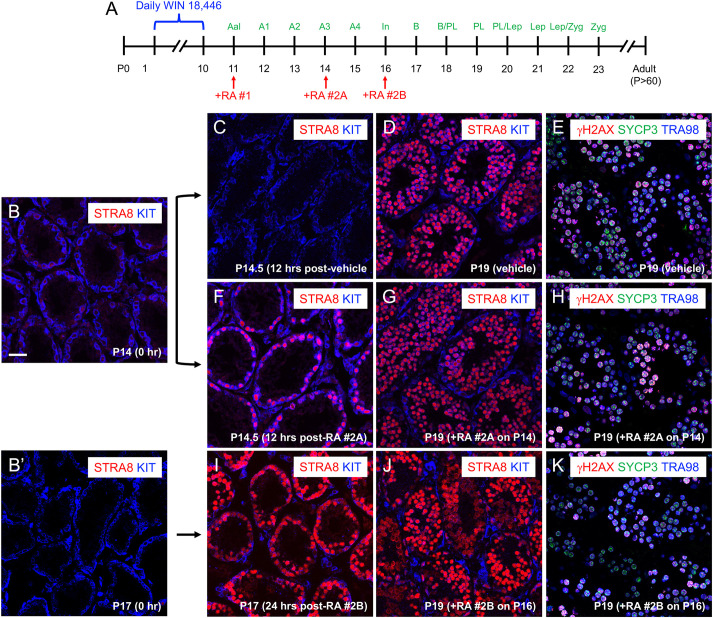
**Precocious RA does not advance the timing of meiotic initiation.** (A) Spermatogenesis was synchronized on this timeline, and a dose of 10 µl of exogenous RA (10 µg/µl) in DMSO was injected to all mice on P11 to induce spermatogonia differentiation (+RA #1). Groups of mice then received either vehicle or a second dose of RA at P14 (+RA #2A, A_3_ spermatogonia) or P16 (+RA #2B, In spermatogonia). (B-K) Immunostaining for protein markers (color coding provided on each image) on testis sections from mice that received only RA #1 (C-E), RA #1 plus RA #2A (F-H) or RA #1 plus RA #2B (I-K). (B,B′) Immunostaining before any treatments. Scale bar: 25 µm. Each experiment was repeated thrice and *n*≥4 mice were used for each experiment.

The current model for male germ cell meiosis postulates that initiation is triggered by rising RA levels, which occur in the normal testis at stages VII-VIII of the seminiferous epithelium (Endo et al., 2015; Hogarth et al., 2014). To test this model, we employed a gain-of-function approach by injecting RA into mice with synchronized spermatogenesis whose testes contained KIT^+^ spermatogonia near the middle and at late stages of differentiation (Fig. 4A-B′). We predicted that if these differentiating spermatogonia were both responsive to and awaiting the signal provided by RA, then they should activate STRA8 and precociously enter meiosis. Differentiating spermatogonia at P14 (A_3_) and P16 (In/B) were indeed RA responsive, as evidenced by activation of STRA8 in response to exogenous RA (100 µg), as expected (Fig. 4C versus 4F,I). By P19, testes from mice that were given vehicle or the second pulse of RA (at P14 or P16, respectively) all contained STRA8^+^ preleptotene spermatocytes, revealing that the timing of meiotic initiation was not advanced, and thus RA was not a limiting factor for the initiation of meiosis spermatogonia at mid (A_3_, Fig. 4G,H) and late (In/B, Fig. 4J,K) stages of differentiation.

### RA was dispensable for spermatogonia differentiation and meiotic initiation *in vitro*

**Fig. 5.**
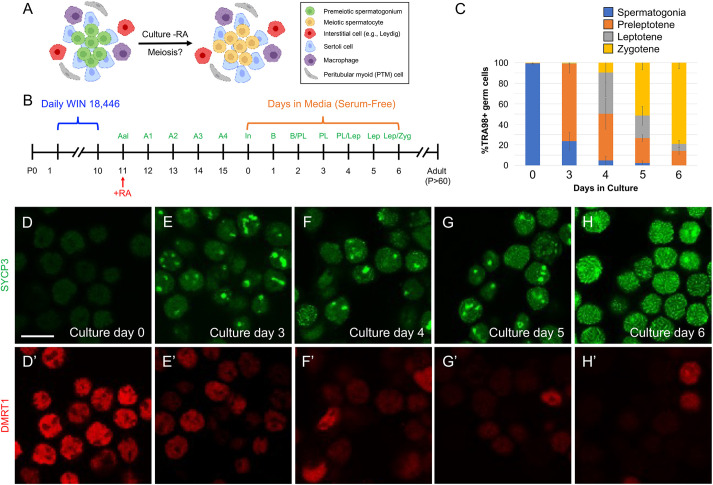
**Spermatogonia complete differentiation and initiate meiosis in retinoid-free media *in vitro*.** (A,B) Mice with synchronized spermatogenesis were given a single injection of RA on P11 and euthanized on P16. Single cell testis suspensions were maintained in serum-free media for 6 days. Cultures were harvested 12 h after plating (day 0) and then on subsequent days. (C) Quantitation of the identity of TRA98^+^ germ cells based on characteristic localization patterns of SYCP3 and DMRT1. (D-H′) Immunostaining was carried out on fixed cells using antibodies against SYCP3 (green) and DMRT1 (red). The day of culture is indicated on each image. Scale bar: 25 µm. Each experiment was repeated thrice and *n*≥4 mice were used for each experiment.

We next sought to verify these *in vivo* results by examining meiotic initiation and progression *in vitro*, where RA levels are readily manipulable. Testis single cell suspensions, in which tubule architecture was disrupted (Fig. 5A), were isolated 5 days after *in vivo* RA-induced differentiation, when differentiating (In/B) spermatogonia were the most advanced germ cell type (Fig. 5B). Cultures were maintained in serum-free media lacking retinoids and were harvested on successive days for immunostaining with established fate markers of spermatogonia (DMRT1^+^/TRA98^+^) and meiosis progression (DMRT1^−^/SYCP3^+^). At P16 (day 0 of culture), 99.3% of TRA98^+^/DMRT1^+^/SYCP3^−^ germ cells in single cell suspensions were spermatogonia (Fig. 5C-D′), while somatic Sertoli cells were TRA98^−^/DMRT1^+^/SYCP3^−^ (Lei et al., 2007). By P19 (3 days in culture), only 23.6% were spermatogonia, and 75.2% had become preleptotene spermatocytes, as expected (Fig. 5C,E,E′). On P20 (4 days in culture), 45.5% were in preleptonema and 40.1% were in leptonema (Fig. 5C,F,F′). On P21 (5 days in culture), 21.8% were in leptonema and 51.6% were in zygonema (Fig. 5C,G,G′). On P22 (6 days in culture), 79.0% were in zygonema/pachynema (Fig. 5C,H,H′). Collectively, male germ cells were able to enter and progress through meiosis in serum-free media lacking retinoids.

### The meiotic gene expression program was largely unaltered in RA-deficient testes

**Fig. 6.**
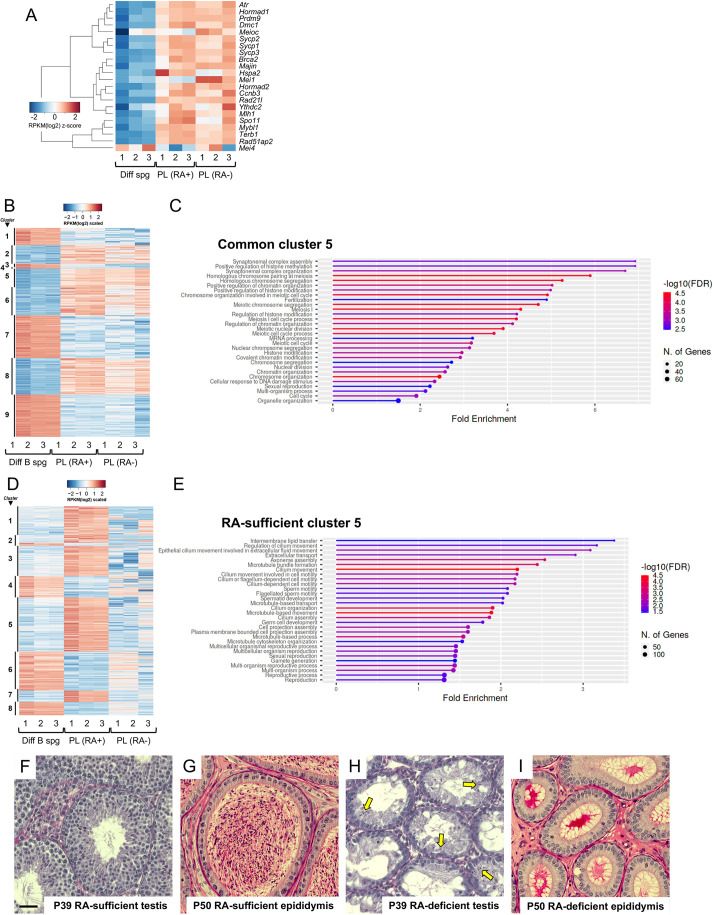
**Gene expression changes in RA-deficient germ cells reveal a requirement for the second pulse of RA in completion of spermiogenesis.** (A) Heatmap showing log-normalized mRNA abundance for a panel of meiotic genes in the noted samples (Diff spg, In/B spermatogonia, P17; PL, preleptotene spermatocytes, P19; RA+, RA sufficient; RA−, RA deficient) according to the scale. (B) Heatmap showing scaled mRNA abundance of significantly differentially expressed genes between differentiating B spermatogonia and preleptotene spermatocytes from both RA-sufficient and RA-deficient testes. Abundance is indicated according to the color scale and unsupervised gene clusters are shown on the left. (C) Lollipop plot indicates the Gene Ontology Enrichment Analysis of genes from cluster 5 in B. (D) Heatmap showing scaled mRNA abundance of significantly differentially expressed genes between differentiating B spermatogonia and preleptotene spermatocytes from both RA-sufficient and RA-deficient testes. Abundance is indicated according to the color scale and unsupervised gene clusters are shown on the left. (E) Lollipop plot indicates the Gene Ontology Enrichment Analysis of genes from cluster 5 in D. (F-I) Periodic acid-Schiff stained sections from RA-sufficient (F,G) or RA-deficient (H,I) testis and epididymis, with ages indicated. Yellow arrows (in H) indicate the remaining undifferentiated spermatogonia. Scale bar: 50 µm.

A role has been proposed for RA in regulating STRA8-mediated gene expression changes that are crucial for meiotic initiation (Kojima et al., 2019; Gewiss et al., 2021). However, STRA8 is not strictly required for meiotic initiation (Baltus et al., 2006; Mark et al., 2008); our data presented here reveal that, after a single transient dose of RA to induce differentiation, male germ cells initiated and completed meiosis ∼8 days later in the absence of RA. We therefore examined, during meiotic initiation in preleptonema with and without RA (±RA), germ cell transcriptomes to gauge the requirement for RA in setting the meiotic gene expression program. Transcriptomes of In/B differentiating spermatogonia (EGFP^bright^) and meiotic preleptotene spermatocytes (EGFP^dim^) were identified by RNA-seq using *Uchl1-*EGFP mice with synchronized spermatogenesis as germ cells transitioned ±RA from mitosis to meiosis. As expected, there were dramatic changes (largely increases) in the abundance of numerous key meiotic mRNAs, as RA-sufficient differentiating spermatogonia initiated meiosis as preleptotene spermatocytes (Fig. 6A). However, in preleptotene spermatocytes from RA-deficient versus RA-sufficient testes, these mRNAs exhibited minimal differences in their abundance (Fig. 6A).

We next assessed other genes with mRNA levels that were also unchanged in the absence of RA. The abundance of 2497 mRNAs changed similarly when comparing In/B differentiating spermatogonia and preleptotene spermatocytes from either RA-sufficient or RA-deficient testes (Fig. 6B, Table S6). In agreement with our targeted analysis (Fig. 6B), genes in ‘common cluster 5′, which had significantly elevated mRNA levels in both (±RA) preleptotene spermatocyte groups, exhibited significant over-representation of genes associated with meiosis GO terms (Fig. 6C, Table S7). These data reinforce our observations, based on cellular morphology and expression of established markers of germ cell identity, that male germ cells similarly initiated meiosis in both RA-sufficient and -deficient testes.

### Male germ cells in RA-deficient testes failed to complete spermiogenesis and produce sperm

To elucidate the biological role of RA at the time of meiotic initiation, we identified the 4768 genes with significantly differential mRNA abundance in RA-sufficient preleptotene spermatocytes versus differentiating In/B spermatogonia that were significantly changed in RA-deficient preleptotene spermatocytes (Table S6). Interestingly, genes in ‘RA-sufficient cluster 5’, which were induced in RA-sufficient but not RA-deficient preleptotene spermatocytes, contained a significant over-representation of spermiogenesis genes, including those involved in cilia function, axoneme assembly, flagellar motility, spermatid development and gamete development (Fig. 6D,E, Table S8).

Owing to these significant yet rather unexpected changes in expression of spermiogenesis genes, we assessed whether spermiogenesis was successfully completed in RA-deficient testes, using the well-documented length of each phase of spermatogenesis and spermiogenesis (Leblond and Clermont, 1952a,b; Roosen-Runge, 1952; Oakberg, 1956b) as a temporal guide. As expected, P39 RA-sufficient testes contained numerous condensing spermatids (Fig. 6F), and at P50, RA-sufficient epididymides were filled with sperm (Fig. 6G). In contrast, P39 RA-deficient testes contained spermatogonia and few condensing spermatids with highly misshapen heads (Fig. 6H) and, as a consequence, RA-deficient P50 epididymides were empty, with no apparent sperm (Fig. 6I). In conclusion, the pulse of RA at meiotic initiation is required for normal completion of spermiogenesis and the formation of testicular sperm.

## DISCUSSION

The results presented here reveal the signal provided by RA is not a requisite checkpoint for meiotic initiation in the testis as previously proposed (Bowles et al., 2006; Bowles and Koopman, 2007; Griswold et al., 2012; Koubova et al., 2006; van Pelt and de Rooij, 1990). Instead, the primary role of RA in the male in terms of meiosis is to initiate the lengthy preceding program of spermatogonia differentiation. It is during this essential differentiation program that gene expression changes occur that are sufficient for the initiation of, progression through and completion of meiosis. In the absence of RA, the meiotic gene expression program was largely unaltered and male germ cells entered and completed meiosis, forming haploid spermatids that were unable to complete spermiogenesis.

It has been known for nearly 100 years that vitamin A, and thus RA, are essential for normal spermatogenesis and male fertility (Wolbach and Howe, 1925; Mason, 1933). Until recently, loss-of-function approaches to deplete testicular RA were carried out by feeding rodents a vitamin A-deficient (VAD) diet. VAD testes contained only spermatogonia and, in some studies, limited numbers of preleptotene spermatocytes (Mason, 1933; Mitranond et al., 1979; Unni et al., 1983; van Pelt et al., 1995). Animals with VAD that were re-fed vitamin A or injected with either retinol or RA recovered full spermatogenesis (Huang et al., 1990; Mason, 1933; Morales and Griswold, 1987; van Pelt and de Rooij, 1990, 1991). This resumption of spermatogenesis occurred in a synchronized fashion (van Pelt and de Rooij, 1990), and led to restoration of fertility. However, VAD often caused incomplete RA deficiency – in one study, 93% (14/15) of VAD rats had testes containing numerous meiotic spermatocytes (van Pelt and de Rooij, 1991). In more recent experimental approaches, researchers depleted RA using the potent and selective RA synthesis inhibitor WIN 18,446 (used in this study) (Arnold et al., 2015a,b). Careful analyses of WIN 18,446-treated animals revealed an initial and essential requirement for RA at spermatogonial differentiation (formation of A_1_ spermatogonia) (Hogarth et al., 2013). Therefore, as differentiation requires RA, its subsequent roles in processes such as meiosis, spermiogenesis and spermiation in males have thus far remained unexamined.

In lower organisms such as yeast, meiosis initiates downstream of a carbon substrate switch – from glucose to acetate – in response to the aptly named ‘inducer of meiosis’ (IME1; Smith et al., 1993; Mandel et al., 1994). This master regulator of meiotic initiation is recruited to meiotic gene promoters to activate their transcription. In mammals, an analogous role has been proposed for STRA8, which is activated in A_1_ differentiating spermatogonia and again in preleptotene spermatocytes by RA (Oulad-Abdelghani et al., 1996; Koubova et al., 2006; Anderson et al., 2008; Zhou et al., 2008). However, a conserved role is unlikely for three key reasons. First, STRA8 does not resemble the pioneering transcription factor IME1, as STRA8 lacks a specific binding preference for meiosis-specific gene promoters, and *Stra8* KO spermatocytes had relatively few gene-specific changes (Kojima et al., 2019). Second, although *Stra8* KO germ cells in congenic C57BL/6 mice arrest during preleptonema (Baltus et al., 2006; Anderson et al., 2008), those in a mixed genetic background progress through meiosis for up to another week, making it to zygonema and even topachynema (Mark et al., 2008). These disparate phenotypes suggest the existence of genetic modifier(s) of STRA8 in the C57Bl/6 background. As STRA8 is evolutionarily conserved in all mammals, it is difficult to conceive that it might serve as the meiotic gatekeeper only in one specific background of mice. Third, we showed here that male germ cells initiated, progressed through and completed meiosis without RA (and thus detectable STRA8) during preleptonema. However, in our treatment paradigm, male germ cells clearly expressed STRA8, albeit transiently, 8 days before meiosis, at the initiation of differentiation. This likely explains how the phenotype of germ cells in RA-deficient testes differs from that of *Stra8* KO germ cells, which arrest in both backgrounds by at least pachynema.

In rodent testes, four crucial cell transitions – spermatogonial differentiation, meiotic initiation, spermatid elongation and spermiation – are spatially adjacent and occur within seminiferous epithelium stages VII-IX (Leblond and Clermont, 1952a; Oakberg, 1956a,b). There is some evidence in the literature for the dependency of each of these transitions upon the rise in RA levels, which occurs at stages VII-VIII (Endo et al., 2017; Hogarth et al., 2014). However, these events are not similarly colocalized in testes from other species, including humans (Muciaccia et al., 2013). Based on this, it is difficult to imagine how discrete changes in RA levels would play key conserved roles in regulation of those four events in species with seminiferous epithelia lacking co-linear organization.

The fetal gonad has long been employed as a model system for studying germ cell entry into meiosis I, which occurs in the ovary but not the testis (McLaren, 1983, 1984; McLaren and Southee, 1997). In 2006, two studies reported that RA provides the ‘meiosis-inducing substance’ (MIS) in the ovary, and that its action is prevented in the testis by the catabolic enzyme CYP26B1, which serves as the ‘meiosis-preventing substance’ (MPS) (Bowles et al., 2006; Koubova et al., 2006). This conclusion was called into question in a report from the Duester laboratory in 2011 (Kumar et al., 2011), which examined meiotic initiation in fetal gonads of *Aldh1a2* and *Aldh1a2/Aldh1a3* KO mice. *Aldh1a2* and *Aldh1a3* encode two out of the three retinaldehyde dehydrogenases (excepting *Aldh1a1*), thus KO fetal gonads had greatly reduced levels of RA. However, despite this reduction in RA, *Aldh1a2/Aldh1a3* KO gonads at E13.5 (when female germ cells enter meiosis in preleptonema) contained both abundant *Stra8* mRNA and γH2AX^+^ oocytes (the latter indicating that DSBs had formed, which implies entry into meiotic prophase). Here, in RA-deficient testes, there were also no significant changes in *Stra8* mRNA levels in preleptonema (Table S6), although STRA8 protein was completely undetectable (Fig. 2C, Fig. S2B). Although a pulse of RA increased both *Stra8* mRNA levels in A_diff_ spermatogonia (Table S1) and its encoded protein in both A_diff_ spermatogonia and preleptotene spermatocytes (Figs 1C, 4F,I, Table S1), *Stra8* steady-state mRNA levels in preleptotene spermatocytes appear to be RA independent.

In the fetal ovary, the differentiation of oogonia and subsequent meiotic entry of preleptotene oocytes have not been temporally isolated because they occur in rapid succession. This contrasts with the differentiation program in mouse testes, which is 8.6 days. This difference makes some sense biologically, as the mammalian female germline is not a stem cell-based system, as it is in the male, and relies on proliferation of spermatogonia to generate millions of new gametes daily. Thus, it is possible that RA serves a similar role in the ovary as in the testis: to drive commitment to a differentiation program that culminates, without a requisite second exposure to RA, in meiosis. This difference perhaps helps to explain the seemingly contradictory conclusions regarding the role of RA as the ‘meiosis-inducing substance’ (Bowles et al., 2006; Griswold et al., 2012; Koubova et al., 2006; Kumar et al., 2011; Teletin et al., 2019). As *Aldh1a2* deletion caused lethality at ∼E9.25 (Mic et al., 2002, 2003; Niederreither et al., 1999), embryos were ‘rescued’ by maternal dietary RA supplementation (Kumar et al., 2011). This RA supplementation certainly could have supported the differentiation of oogonia and – as observed here – once germ cells differentiated in response to RA, a second pulse was unnecessary for initiation of and progression through meiosis. This premise is also supported by a study using germ cell conditional KO *Aldh1a1-Aldh1a3* mice. Using these mice and treatment with WIN 18,446, it was concluded that higher RA levels are required for STRA8 activation and initiation of differentiation versus meiosis (Teletin et al., 2019). Thus, a unifying theme might be reached by changing the role of RA from ‘meiosis-inducing substance’ to germ cell ‘differentiation-inducing substance’.

As the ‘differentiation-inducing substance’, RA directs a commitment to meiosis. This commitment during spermatogonial differentiation is supported by our transcriptome analyses, which revealed that subsets of mRNAs encoding proteins with established roles in meiosis were upregulated during differentiation. This reveals that, in the days before meiotic initiation, preparations were already under way to generate the gene products necessary for the meiotic program. This meshes with recently published work from the Namekawa laboratory, which identified DNA regions specific to meiotic genes containing clusters of transcriptional enhancers (termed super-enhancers or SEs). Interestingly – and with direct relevance to the results presented here – these meiotic SEs were enriched in spermatogonia with H3K4me3 and H3K27ac (Maezawa et al., 2020). These histone modifications are characteristic of genes poised for transcription and reveal that preparation for meiosis occurs well in advance (∼8.6 days) of meiosis. The observation here that the spermatogonia that differentiated in response to RA *in vivo* initiated and proceeded through meiotic prophase in the absence of RA *in vitro*, at the same timeframe as *in vivo*, supports the existence of an intrinsic clock mechanism that preserves a set number of divisions and a rigid timeline for meiosis after RA-induced differentiation. These results highlight the pressing need to expand the study of the enigmatic and understudied differentiation program to uncover mechanisms directing mammalian germ cell commitment to meiosis.

## MATERIALS AND METHODS

### Synchronizing spermatogenesis and blocking retinoic acid (RA) synthesis *in vivo*

All procedures using mice adhered to guidelines outlined in the National Research Council Guide for the Care and Use of Laboratory Animals and were approved by the Animal Care and Use Committee at East Carolina University (approval A3469-01). *Uchl1-eGfp* mice were obtained from The Jackson Laboratory (stock 22476). *Uchl1-eGfp* mice were outcrossed with CD-1 (Charles River Laboratories, stock 022) to increase litter size and reduce dam cannibalism (Kirsanov et al., 2022). The day of birth was designated as P0. Spermatogenesis was synchronized as recently reported (Kirsanov et al., 2022). Briefly, mice were administered the RA synthesis inhibitor bis-(dichloroacetyl)-diamine/WIN 18,446 (Cayman Chemical, 14018) at 100 µg/g body weight in vehicle (dimethyl sulfoxide, DMSO). Compound was fed daily from P1 to P10 with a 24-gauge feeding needle. At P11, 10 µl of RA (10 µg/µl) in DMSO was injected subcutaneously to initiate differentiation. For RA-sufficient testes, mice were allowed to age without further compound administration. For RA-deficient testes, WIN 18,446 was administered daily until euthanasia. Mice were humanely euthanized before P7 by decapitation, and after P7 by asphyxiation in CO_2_ followed by cervical dislocation.

### Fluorescence-activated cell sorting

EGFP^+^ germ cells from *Uchl1-eGfp* mice were sorted on a Becton Dickinson AriaFusion cell sorter as described previously (Kirsanov et al., 2022). Briefly, a 100 mW 488 nm laser was used for excitation of the EGFP signal and a 530/30 bandpass filter was used for detection of the emitted fluorescence. Dead cells were removed using forward and side scatter gating, and fluorescence gating on cells excluding DAPI. Single DNA-containing cells were identified using a DRAQ5 fluorescent probe (Thermo Fisher Scientific, 62251). Doublets were removed using FSC-height versus FSC-area plots. An 85 µm nozzle was used for sorting, and the flow rate was controlled to 5000-9000 events s^−1^. Cells were recovered in sorting buffer (15% FBS+10 mM EDTA+10 mM HEPES in 1×PBS) in 5 ml polypropylene tubes (Corning LS) pre-coated with 10% BSA. Cell purity was assessed by reanalyzing a small aliquot of the sorted cells and by immunostaining both testes from treated mice and sorted cells with cell fate markers (Kirsanov et al., 2022).

### *In vitro* cell cultures and immunostaining

Single cell suspensions were prepared and cultured as described previously (Kirsanov et al., 2022). Briefly, testes were detunicated in Hanks' Balanced Salt Solution (HBSS, Thermo Fisher Scientific, 14170120) and transferred to a solution containing 4.5 ml of 0.25% trypsin (Thermo Fisher Scientific, 150065) and 0.5 ml DNase1 (7 mg/ml, Sigma-Aldrich, DN25) in HBSS at 37°C for 3 min. Another 1 ml DNase1 in HBSS was added for a 1 min incubation at 37°C. Trypsin was deactivated, uniquely here, with 1 ml charcoal-stripped fetal bovine serum (FBS, Thermo Fisher Scientific, A33821201) lacking retinoids. The cell mixture was filtered through a 40 μm sieve and centrifuged at 600 ***g***. Cell pellets were resuspended in serum-free DMEM/F12 with Glutamax (Thermo Fisher Scientific, 10565018)+1% penicillin-streptomycin (Thermo Fisher Scientific, 15070063). For *in vitro* culture, 175,000 cells were seeded into each well of a glass-bottomed 96-well dish (Cellvis P96-1.5H-N). Media were made fresh and changed daily, and cultures were maintained at 34°C in 5% CO_2_. For the gain-of-function experiments, RA was added at 1 µM final concentration.

At the end of each experiment, cells were fixed in the dish with 4% PFA for 10 min and then washed thrice with 1×PBS. Cells were permeabilized with 1×PBS+0.1% Triton X-100 and immunostaining performed as described previously (Kirsanov et al., 2022). Fluoroshield (Sigma-Aldrich, #F6057) was diluted 1:10 in 1×PBS+90% glycerol (Sigma-Aldrich, G5516) and 200 µl added to each well; images were obtained using an Olympus Fluoview FV1000 confocal laser-scanning microscope. Each experiment was repeated thrice and *n*≥4 mice were used for each experiment.

### Meiotic chromosome spreads

Meiotic chromosome spreads were prepared as described previously (Dia et al., 2017). Briefly, detunicated testes were incubated in ice-cold Hypotonic Extraction Buffer [HEB; 30 mM Tris HCl, 50 mM sucrose, 17 mM trisodium citrate dihydrate, 5 mM ethylenediaminetetraacetic acid (EDTA), 0.5 mM dithiothreitol (DTT) and 0.2 mM phenylmethylsulfonyl fluoride (PMSF) at pH 8.2] for 30-60 min. Testes were separated into 3 mm chunks that were minced in 100 mM sucrose (pH 8.2). Slides were placed in a Coplin jar containing 1% paraformaldehyde and 0.15% Triton X-100 in 1×PBS (pH 9.2) for 1 h at room temperature. Slides were dried and washed with 0.4% Photo-Flo 200 (Eastman Kodak) in 1×PBS twice for 5 min each, then with 0.4% Photo-Flo 200 in H_2_O once for 5 min. Slides were air-dried and stored at −80°C. Immunostaining was carried out using standard methods (Niedenberger and Geyer, 2018) and as described below, and images were obtained using an Olympus Fluoview FV1000 confocal laser-scanning microscope.

### Histology and indirect immunofluorescence

For histological analyses, whole testes were immersion-fixed in Bouin's solution or PFA as previously described (Niedenberger et al., 2015). For histological analyses, 5 µm Bouin's-fixed sections were stained with periodic acid-Schiff using standard methods, and images captured on an Axio Observer A1 microscope with an Axiocam 503 color digital camera and Zen software (Carl Zeiss Microscopy). Indirect immunofluorescence (IIF) was carried out on cryosections as previously described (Niedenberger and Geyer, 2018). Primary antibodies were: anti-TRA98 (1:1000, Abcam, ab82527), anti-GFRA1 (1:800, R&D Systems, AF-560), anti-KIT (1:1000, R&D Systems, AF-1356) anti-ZBTB16/PLZF (1:400, R&D Systems, AF-294), anti-H1F6 (1:500; Inselman et al., 2003), anti-DMRT1 (1:1000; Lei et al., 2007), anti-STRA8 (1:3000, Abcam, ab49602) and anti-γH2AX (1:400, Abcam, ab11174). Primary antibody was omitted in negative controls. Secondary antibodies were all diluted 1:500: Alexa Fluor donkey anti-rabbit-488 (Thermo Fisher Scientific, A21206); Alexa Fluor donkey anti-goat-488, (Thermo Fisher Scientific, A11055); Alexa Fluor donkey anti-goat-555 (Thermo Fisher Scientific, A32816); Alexa Fluor donkey anti-rat-555 (Thermo Fisher Scientific, A48270). Fluorescently conjugated anti-SYCP3 (anti-SYCP3-488, 1:200, Abcam, ab205846) and anti-lectin (lectin-488, 1:500, Thermo Fisher Scientific L32470; lectin-594, 1:500 Thermo Fisher Scientific L32471) antibodies were also used. Coverslips were mounted with Fluoroshield (Sigma-Aldrich, F6057), and images obtained using an Olympus Fluoview FV1000 confocal laser-scanning microscope (Olympus America). Each experiment was repeated thrice and *n*≥4 mice were used for each experiment.

### DNA content measurement for DNA ploidy

Aliquots of testis single cell suspensions (1×10^6^ cells/ml) were first incubated with Trypan Blue to identify percentages of dead cells (Strober, 2015). The rest of the suspension was immediately fixed in ice-cold 70% ethanol and stored at −20°C. On the day of the experiment, ethanol-suspended cells were washed twice in 1×PBS and finally resuspended in 300 µl of 20 µg/ml propidium iodide (PI)/Triton X-100 staining solution with 5 µg/ml RNase A at room temperature for 30 min. DNA content measurements were performed using a 5-laser Cytek Aurora flow cytometer equipped with a 50 mW 561 laser. For PI emission, the YG4 band pass (661/18) was used. Flow rate was set to low to achieve the best quality, which was evident from the very low coefficient of variation (CV) values of the peaks (indicating peaks were generated from cells with the same DNA content). Data were analyzed using a classical linear scale. Testicular single cell suspensions from adult wild-type CD-1 testes were used for calibration and as positive controls.

### Quantitative (q)RT-PCR

Mice with synchronized spermatogenesis were euthanized at P17 (RA-sufficient In/B spermatogonia) and at P19 (RA-sufficient or RA-deficient preleptotene spermatocytes). FACS was used to isolate 2-3×10^6^ In/B spermatogonia and 2-3×10^6^ preleptotene spermatocytes (three or four males per litter). Total RNA was isolated from three independent preparations using the GeneJet RNA purification kit (Thermo Fisher Scientific, K0702). For qRT-PCR analyses, 0.5 μg total RNA was reverse-transcribed using the iScript cDNA Synthesis Kit (Bio-Rad, 1725037). qPCR was performed using the SsoFast EvaGreen Supermix (Bio-Rad, 1725201) on the Bio-Rad CFX-384 or CFX-96 real-time PCR System. Gene expression was normalized to the average *C*_t_ values of the housekeeping gene *Actb* and expressed as 2^−Δ^*^C^*_t_. Primer sequences used were: *Stra8* forward, 5′-GAGGTCAAGGAAGAATATGC; *Stra8* reverse, 3′-CAGAGACAATAGGAAGTGTC; *Rec8* forward, 5′-CTACCTAGCTTGCTTCTTCC; *Rec8* reverse, 5′-GCCTCTAAAAGGTGTCGAA; *Dmc1* forward, 5′-CCCTCTGTGTGACAGCTCAAC; *Dmc1* reverse, 5′-GGTCAGCAATGTCCCGAAG; *Actb* forward, 5′-TCCGATGCCCTGAGGCTCTTTTC; *Actb* reverse, 5′-CTTGCTGATCCACATCTGCTGGAA.

### RNA-sequencing

FACS-isolated *Uchl1-*EGFP^+^ germ cells were used for total RNA isolation from two or three independent cell preparations using the GeneJet RNA purification kit (Thermo Fisher Scientific, K0702). Subsequently, cDNA libraries were produced from total RNA with the TruSeq Stranded mRNA kit (Illumina, 20020594) according to the manufacturer's recommendations. Libraries were sequenced with an Illumina NovaSeq6000 instrument with single-end (75 bp) chemistry to ∼30 M/sample depth. Adapter of RNA-seq reads were trimmed with cutadapt(v1.9) and reads with quality less than 20 were removed. STAR(v2.5.2b) was used to map the reads to reference mm9 with --outFilterMismatchNoverLmax 0.04. Reads were then annotated with featureCounts(v1.5.1) to gene level. DESeq2 was used to determine differentially expressed genes (DEGs) based on Log2Fold-Change >1 and an adjusted *P*-value <0.05. Separate pairwise DEG comparisons were performed (Table S1). DEGs were used to produce heatmaps with the package ‘pheatmap’ (v1.0.12) in R (v4.1.0) with genes clustered on complete linkage based on the Euclidean distance. Gene clusters in each heatmap were identified by *k*-means, and genes in individual smaller clusters further combined to generate gene lists representing the major clusters in each heatmap. Gene ontology enrichment analysis was performed with ShinyGo v0.75 with default parameters and background normalization (Ge et al., 2020) and AmiGO2 Biological pathways V2017.5 (Carbon et al., 2009).

### Statistics

Statistical differences between experimental groups were determined using one-way ANOVA and Student's *t*-test, with significance levels set at *P*<0.05. Error bars show one standard deviation.

## Supplementary Material

10.1242/develop.201638_sup1 Supplementary information

## References

[DEV201638C1] Anderson, E. L., Baltus, A. E., Roepers-Gajadien, H. L., Hassold, T. J., De Rooij, D. G., Van Pelt, A. M. M. and Page, D. C. (2008). Stra8 and its inducer, retinoic acid, regulate meiotic initiation in both spermatogenesis and oogenesis in mice. *Proc. Natl. Acad. Sci. USA* 105, 14976-14980. 10.1073/pnas.0807297105 18799751 PMC2542382

[DEV201638C2] Arnold, S. L. M., Kent, T., Hogarth, C. A., Griswold, M. D., Amory, J. K. and Isoherranen, N. (2015a). Pharmacological inhibition of ALDH1A in mice decreases all-trans retinoic acid concentrations in a tissue specific manner. *Biochem. Pharmacol.* 95, 177-192. 10.1016/j.bcp.2015.03.001 25764981 PMC4420653

[DEV201638C3] Arnold, S. L., Kent, T., Hogarth, C. A., Schlatt, S., Prasad, B., Haenisch, M., Walsh, T., Muller, C. H., Griswold, M. D., Amory, J. K. et al. (2015b). Importance of ALDH1A enzymes in determining human testicular retinoic acid concentrations. *J. Lipid Res.* 56, 342-357. 10.1194/jlr.M054718 25502770 PMC4306688

[DEV201638C4] Baltus, A. E., Menke, D. B., Hu, Y.-C., Goodheart, M. L., Carpenter, A. E., De Rooij, D. G. and Page, D. C. (2006). In germ cells of mouse embryonic ovaries, the decision to enter meiosis precedes premeiotic DNA replication. *Nat. Genet.* 38, 1430-1434. 10.1038/ng1919 17115059

[DEV201638C5] Bowles, J. and Koopman, P. (2007). Retinoic acid, meiosis and germ cell fate in mammals. *Development* 134, 3401-3411. 10.1242/dev.001107 17715177

[DEV201638C6] Bowles, J., Knight, D., Smith, C., Wilhelm, D., Richman, J., Mamiya, S., Yashiro, K., Chawengsaksophak, K., Wilson, M. J., Rossant, J. et al. (2006). Retinoid signaling determines germ cell fate in mice. *Science* 312, 596-600. 10.1126/science.1125691 16574820

[DEV201638C7] Carbon, S., Ireland, A., Mungall, C. J., Shu, S., Marshall, B., Lewis, S., the AmiGO Hub and the Web Presence Working Group. (2009). AmiGO: online access to ontology and annotation data. *Bioinformatics* 25, 288-289. 10.1093/bioinformatics/btn615 19033274 PMC2639003

[DEV201638C8] Culty, M. (2013). Gonocytes, from the fifties to the present: is there a reason to change the name? *Biol. Reprod.* 89, 46. 10.1095/biolreprod.113.110544 23843237

[DEV201638C9] Dia, F., Strange, T., Liang, J., Hamilton, J. and Berkowitz, K. M. (2017). Preparation of meiotic chromosome spreads from mouse spermatocytes. *J. Vis. Exp.* 129, e55378. 10.3791/55378 PMC5755458 29286440

[DEV201638C10] Endo, T., Romer, K. A., Anderson, E. L., Baltus, A. E., De Rooij, D. G. and Page, D. C. (2015). Periodic retinoic acid-STRA8 signaling intersects with periodic germ-cell competencies to regulate spermatogenesis. *Proc. Natl. Acad. Sci. USA* 112, E2347-E2356. 10.1073/pnas.1505683112 25902548 PMC4426408

[DEV201638C11] Endo, T., Freinkman, E., De Rooij, D. G. and Page, D. C. (2017). Periodic production of retinoic acid by meiotic and somatic cells coordinates four transitions in mouse spermatogenesis. *Proc. Natl. Acad. Sci. USA* 114, E10132-E10141. 10.1073/pnas.1710837114 29109271 PMC5703301

[DEV201638C12] Ge, S. X., Jung, D. and Yao, R. (2020). ShinyGO: a graphical gene-set enrichment tool for animals and plants. *Bioinformatics* 36, 2628-2629. 10.1093/bioinformatics/btz931 31882993 PMC7178415

[DEV201638C13] Gewiss, R. L., Shelden, E. A. and Griswold, M. D. (2021). STRA8 induces transcriptional changes in germ cells during spermatogonial development. *Mol. Reprod. Dev.* 88, 128-140. 10.1002/mrd.23448 33400349 PMC7920925

[DEV201638C14] Griswold, M. D., Hogarth, C. A., Bowles, J. and Koopman, P. (2012). Initiating meiosis: the case for retinoic acid. *Biol. Reprod.* 86, 35. 10.1095/biolreprod.111.096610 22075477 PMC3290665

[DEV201638C15] Haneji, T., Maekawa, M. and Nishimune, Y. (1983). Retinoids induce differentiation of type A spermatogonia in vitro: organ culture of mouse cryptorchid testes. *J. Nutr.* 113, 1119-1123. 10.1093/jn/113.6.1119 6133923

[DEV201638C16] Hogarth, C. A., Evanoff, R., Mitchell, D., Kent, T., Small, C., Amory, J. K. and Griswold, M. D. (2013). Turning a spermatogenic wave into a tsunami: synchronizing murine spermatogenesis using WIN 18,446. *Biol. Reprod.* 88, 40. 10.1095/biolreprod.112.105346 23284139 PMC3589231

[DEV201638C17] Hogarth, C. A., Arnold, S., Kent, T., Mitchell, D., Isoherranen, N. and Griswold, M. D. (2014). Processive pulses of retinoic acid propel asynchronous and continuous murine sperm production. *Biol. Reprod.* 92, 1-11. 10.1095/biolreprod.114.126326 PMC4326729 25519186

[DEV201638C18] Huang, H. F. S., Marshall, G. R. and Nieschlag, E. (1990). Enrichment of the stages of the seminiferous epithelium in vitamin A-replaced-vitamin A-deficient rats. *J. Reprod. Fertil.* 88, 51-60. 10.1530/jrf.0.0880051 2313653

[DEV201638C19] Inselman, A., Eaker, S. and Handel, M. A. (2003). Temporal expression of cell cycle-related proteins during spermatogenesis: establishing a timeline for onset of the meiotic divisions. *Cytogenet Genome Res.* 103, 277-284. 10.1159/000076813 15051948

[DEV201638C20] Kirsanov, O., Johnson, T., Malachowski, T., Niedenberger, B. A., Gilbert, E. A., Bhowmick, D., Ozdinler, P. H., Gray, D. A., Fisher-Wellman, K., Hermann, B. P. et al. (2022). Modeling mammalian spermatogonial differentiation and meiotic initiation in vitro. *Development* 149, dev200713. 10.1242/dev.200713 36250451 PMC9845750

[DEV201638C21] Kojima, M. L., De Rooij, D. G. and Page, D. C. (2019). Amplification of a broad transcriptional program by a common factor triggers the meiotic cell cycle in mice. *eLife* 8, e43738. 10.7554/eLife.43738 30810530 PMC6392498

[DEV201638C22] Koubova, J., Menke, D. B., Zhou, Q., Capel, B., Griswold, M. D. and Page, D. C. (2006). Retinoic acid regulates sex-specific timing of meiotic initiation in mice. *Proc. Natl. Acad. Sci. USA* 103, 2474-2479. 10.1073/pnas.0510813103 16461896 PMC1413806

[DEV201638C23] Koubova, J., Hu, Y.-C., Bhattacharyya, T., Soh, Y. Q. S., Gill, M. E., Goodheart, M. L., Hogarth, C. A., Griswold, M. D. and Page, D. C. (2014). Retinoic acid activates two pathways required for meiosis in mice. *PLoS Genet.* 10, e1004541. 10.1371/journal.pgen.1004541 25102060 PMC4125102

[DEV201638C24] Kumar, S., Chatzi, C., Brade, T., Cunningham, T. J., Zhao, X. and Duester, G. (2011). Sex-specific timing of meiotic initiation is regulated by Cyp26b1 independent of retinoic acid signalling. *Nat. Commun.* 2, 151. 10.1038/ncomms1136 21224842 PMC3034736

[DEV201638C25] Leblond, C. P. and Clermont, Y. (1952a). Definition of the stages of the cycle of the seminiferous epithelium in the rat. *Ann. N. Y. Acad. Sci.* 55, 548-573. 10.1111/j.1749-6632.1952.tb26576.x 13139144

[DEV201638C26] Leblond, C. P. and Clermont, Y. (1952b). Spermiogenesis of rat, mouse, hamster and guinea pig as revealed by the periodic acid-fuchsin sulfurous acid technique. *Am. J. Anat.* 90, 167-215. 10.1002/aja.1000900202 14923625

[DEV201638C27] Lei, N., Hornbaker, K. I., Rice, D. A., Karpova, T., Agbor, V. A. and Heckert, L. L. (2007). Sex-specific differences in mouse DMRT1 expression are both cell type- and stage-dependent during gonad development. *Biol. Reprod.* 77, 466-475. 10.1095/biolreprod.106.058784 17567962 PMC2580730

[DEV201638C28] Maezawa, S., Sakashita, A., Yukawa, M., Chen, X., Takahashi, K., Alavattam, K. G., Nakata, I., Weirauch, M. T., Barski, A. and Namekawa, S. H. (2020). Super-enhancer switching drives a burst in gene expression at the mitosis-to-meiosis transition. *Nat. Struct. Mol. Biol.* 27, 978-988. 10.1038/s41594-020-0488-3 32895557 PMC8690596

[DEV201638C29] Mandel, S., Robzyk, K. and Kassir, Y. (1994). IME1 gene encodes a transcription factor which is required to induce meiosis in Saccharomyces cerevisiae. *Dev. Genet.* 15, 139-147. 10.1002/dvg.1020150204 8205723

[DEV201638C30] Mark, M., Jacobs, H., Oulad-Abdelghani, M., Dennefeld, C., Féret, B., Vernet, N., Codreanu, C.-A., Chambon, P. and Ghyselinck, N. B. (2008). STRA8-deficient spermatocytes initiate, but fail to complete, meiosis and undergo premature chromosome condensation. *J. Cell Sci.* 121, 3233-3242. 10.1242/jcs.035071 18799790

[DEV201638C31] Mason, K. E. (1933). Differences in testis injury and repair after vitamin A-deficiency, vitamin-E deficiency, and inanition. *Am. J. Anat.* 52, 153-239. 10.1002/aja.1000520202

[DEV201638C32] Mccarrey, J. R. (2013). Toward a more precise and informative nomenclature describing fetal and neonatal male germ cells in rodents. *Biol. Reprod.* 89, 47. 10.1095/biolreprod.113.110502 23843236 PMC4076367

[DEV201638C33] McLaren, A. (1983). Studies on mouse germ cells inside and outside the gonad. *J. Exp. Zool.* 228, 167-171. 10.1002/jez.1402280203 6663255

[DEV201638C34] McLaren, A. (1984). Meiosis and differentiation of mouse germ cells. *Symp. Soc. Exp. Biol.* 38, 7-23. 6400220

[DEV201638C35] McLaren, A. and Southee, D. (1997). Entry of mouse embryonic germ cells into meiosis. *Dev. Biol.* 187, 107-113. 10.1006/dbio.1997.8584 9224678

[DEV201638C36] McPhillips, D. M., Kalin, J. R. and Hill, D. L. (1987). The pharmacokinetics of all-trans-retinoic acid and N-(2-hydroxyethyl)retinamide in mice as determined with a sensitive and convenient procedure. Solid-phase extraction and reverse-phase high performance liquid chromatography. *Drug Metab. Dispos.* 15, 207-211. 2882980

[DEV201638C37] Mic, F. A., Haselbeck, R. J., Cuenca, A. E. and Duester, G. (2002). Novel retinoic acid generating activities in the neural tube and heart identified by conditional rescue of Raldh2 null mutant mice. *Development* 129, 2271-2282. 10.1242/dev.129.9.2271 11959834 PMC2833017

[DEV201638C38] Mic, F. A., Molotkov, A., Benbrook, D. M. and Duester, G. (2003). Retinoid activation of retinoic acid receptor but not retinoid X receptor is sufficient to rescue lethal defect in retinoic acid synthesis. *Proc. Natl. Acad. Sci. USA* 100, 7135-7140. 10.1073/pnas.1231422100 12782789 PMC165842

[DEV201638C39] Mitranond, V., Sobhon, P., Tosukhowong, P. and Chindaduangrat, W. (1979). Cytological changes in the testes of vitamin-A-deficient rats: I. Quantitation of germinal cells in the seminiferous tubules. *Acta Anat. (Basel)* 103, 159-168. 10.1159/000145007 419926

[DEV201638C40] Morales, C. and Griswold, M. D. (1987). Retinol-induced stage synchronization in seminiferous tubules of the rat. *Endocrinology* 121, 432-434. 10.1210/endo-121-1-432 3595524

[DEV201638C41] Muciaccia, B., Boitani, C., Berloco, B. P., Nudo, F., Spadetta, G., Stefanini, M., De Rooij, D. G. and Vicini, E. (2013). Novel stage classification of human spermatogenesis based on acrosome development. *Biol. Reprod.* 89, 60. 10.1095/biolreprod.113.111682 23946533

[DEV201638C42] Nau, H. (1986). Species differences in pharmacokinetics and drug teratogenesis. *Environ. Health Perspect.* 70, 113-129. 10.1289/ehp.8670113 3104022 PMC1474298

[DEV201638C43] Niedenberger, B. A. and Geyer, C. B. (2018). Advanced immunostaining approaches to study early male germ cell development. *Stem Cell Res* 27, 162-168. 10.1016/j.scr.2018.01.031 29475796 PMC5894494

[DEV201638C44] Niedenberger, B. A., Busada, J. T. and Geyer, C. B. (2015). Marker expression reveals heterogeneity of spermatogonia in the neonatal mouse testis. *Reproduction* 149, 329-338. 10.1530/REP-14-0653 25737569 PMC4350003

[DEV201638C45] Niederreither, K., Subbarayan, V., Dollé, P. and Chambon, P. (1999). Embryonic retinoic acid synthesis is essential for early mouse post-implantation development. *Nat. Genet.* 21, 444-448. 10.1038/7788 10192400

[DEV201638C46] Oakberg, E. F. (1956a). A description of spermiogenesis in the mouse and its use in analysis of the cycle of the seminiferous epithelium and germ cell renewal. *Am. J. Anat.* 99, 391-413. 10.1002/aja.1000990303 13402725

[DEV201638C47] Oakberg, E. F. (1956b). Duration of spermatogenesis in the mouse and timing of stages of the cycle of the seminiferous epithelium. *Am. J. Anat.* 99, 507-516. 10.1002/aja.1000990307 13402729

[DEV201638C48] Oulad-Abdelghani, M., Bouillet, P., Décimo, D., Gansmuller, A., Heyberger, S., Dollé, P., Bronner, S., Lutz, Y. and Chambon, P. (1996). Characterization of a premeiotic germ cell-specific cytoplasmic protein encoded by Stra8, a novel retinoic acid-responsive gene. *J. Cell Biol.* 135, 469-477. 10.1083/jcb.135.2.469 8896602 PMC2121034

[DEV201638C49] Romer, K. A., De Rooij, D. G., Kojima, M. L. and Page, D. C. (2018). Isolating mitotic and meiotic germ cells from male mice by developmental synchronization, staging, and sorting. *Dev. Biol.* 443, 19-34. 10.1016/j.ydbio.2018.08.009 30149006

[DEV201638C50] Roosen-Runge, E. C. (1952). Kinetics of spermatogenesis in mammals. *Ann. N. Y. Acad. Sci.* 55, 574-584. 10.1111/j.1749-6632.1952.tb26577.x 13139145

[DEV201638C51] Smith, H. E., Driscoll, S. E., Sia, R. A., Yuan, H. E. and Mitchell, A. P. (1993). Genetic evidence for transcriptional activation by the yeast IME1 gene product. *Genetics* 133, 775-784. 10.1093/genetics/133.4.775 8462841 PMC1205399

[DEV201638C52] Strober, W. (2015). Trypan blue exclusion test of cell viability. *Curr. Protoc. Immunol.* 111, A3.B.1-a3.B.3. 10.1002/0471142735.ima03bs111 PMC6716531 26529666

[DEV201638C53] Teletin, M., Vernet, N., Yu, J., Klopfenstein, M., Jones, J. W., Féret, B., Kane, M. A., Ghyselinck, N. B. and Mark, M. (2019). Two functionally redundant sources of retinoic acid secure spermatogonia differentiation in the seminiferous epithelium. *Development* 146, dev170225. 10.1242/dev.170225 30487180 PMC6340151

[DEV201638C54] Unni, E., Rao, M. R. and Ganguly, J. (1983). Histological & ultrastructural studies on the effect of vitamin A depletion & subsequent repletion with vitamin A on germ cells & Sertoli cells in rat testis. *Indian J. Exp. Biol.* 21, 180-192. 6662558

[DEV201638C55] van Pelt, A. M. M. and De Rooij, D. G. (1990). Synchronization of the seminiferous epithelium after vitamin A replacement in vitamin A-deficient mice. *Biol. Reprod.* 43, 363-367. 10.1095/biolreprod43.3.363 2271719

[DEV201638C56] van Pelt, A. M. M. and De Rooij, D. G. (1991). Retinoic acid is able to reinitiate spermatogenesis in vitamin A-deficient rats and high replicate doses support the full development of spermatogenic cells. *Endocrinology* 128, 697-704. 10.1210/endo-128-2-697 1989855

[DEV201638C57] van Pelt, A. M. M., van Dissel-Emiliani, F. M. F., Gaemers, I. C., van der Burg, M. J. M., Tanke, H. J. and de Rooij, D. G. (1995). Characteristics of A spermatogonia and preleptotene spermatocytes in the vitamin A-deficient rat testis. *Biol. Reprod.* 53, 570-578. 10.1095/biolreprod53.3.570 7578681

[DEV201638C58] Velte, E. K., Niedenberger, B. A., Serra, N. D., Singh, A., Roa-DeLaCruz, L., Hermann, B. P. and Geyer, C. B. (2019). Differential RA responsiveness directs formation of functionally-distinct spermatogonial populations at the initiation of spermatogenesis in the mouse. *Development* 146, dev173088. 10.1242/dev.173088 31023878 PMC6602341

[DEV201638C59] Wolbach, S. B. and Howe, P. R. (1925). Tissue changes following deprivation of fat-soluble a vitamin. *J. Exp. Med.* 42, 753-777. 10.1084/jem.42.6.753 19869087 PMC2131078

[DEV201638C60] Xu, H., Beasley, M. D., Warren, W. D., van der Horst, G. T. J. and Mckay, M. J. (2005). Absence of mouse REC8 cohesin promotes synapsis of sister chromatids in meiosis. *Dev. Cell* 8, 949-961. 10.1016/j.devcel.2005.03.018 15935783

[DEV201638C61] Yasvoina, M. V., Genç, B., Jara, J. H., Sheets, P. L., Quinlan, K. A., Milosevic, A., Shepherd, G. M. G., Heckman, C. J. and Özdinler, P. H. (2013). eGFP expression under UCHL1 promoter genetically labels corticospinal motor neurons and a subpopulation of degeneration-resistant spinal motor neurons in an ALS mouse model. *J. Neurosci.* 33, 7890-7904. 10.1523/JNEUROSCI.2787-12.2013 23637180 PMC3963467

[DEV201638C62] Zhou, Q., Nie, R., Li, Y., Friel, P., Mitchell, D., Hess, R. A., Small, C. and Griswold, M. D. (2008). Expression of stimulated by retinoic acid gene 8 (Stra8) in spermatogenic cells induced by retinoic acid: an in vivo study in vitamin A-sufficient postnatal murine testes. *Biol. Reprod.* 79, 35-42. 10.1095/biolreprod.107.066795 18322276 PMC3208264

