# Supplementary material for: Retinoic acid is dispensable for meiotic initiation but required for spermiogenesis in the mammalian testis
Source: Development. 2023 Jul 13;150(14):dev201638. doi: 10.1242/dev.201638 (PMC10357014; doi:10.1242/dev.201638)
Supplement: Supplementary information [file develop-150-201638-s1.pdf]

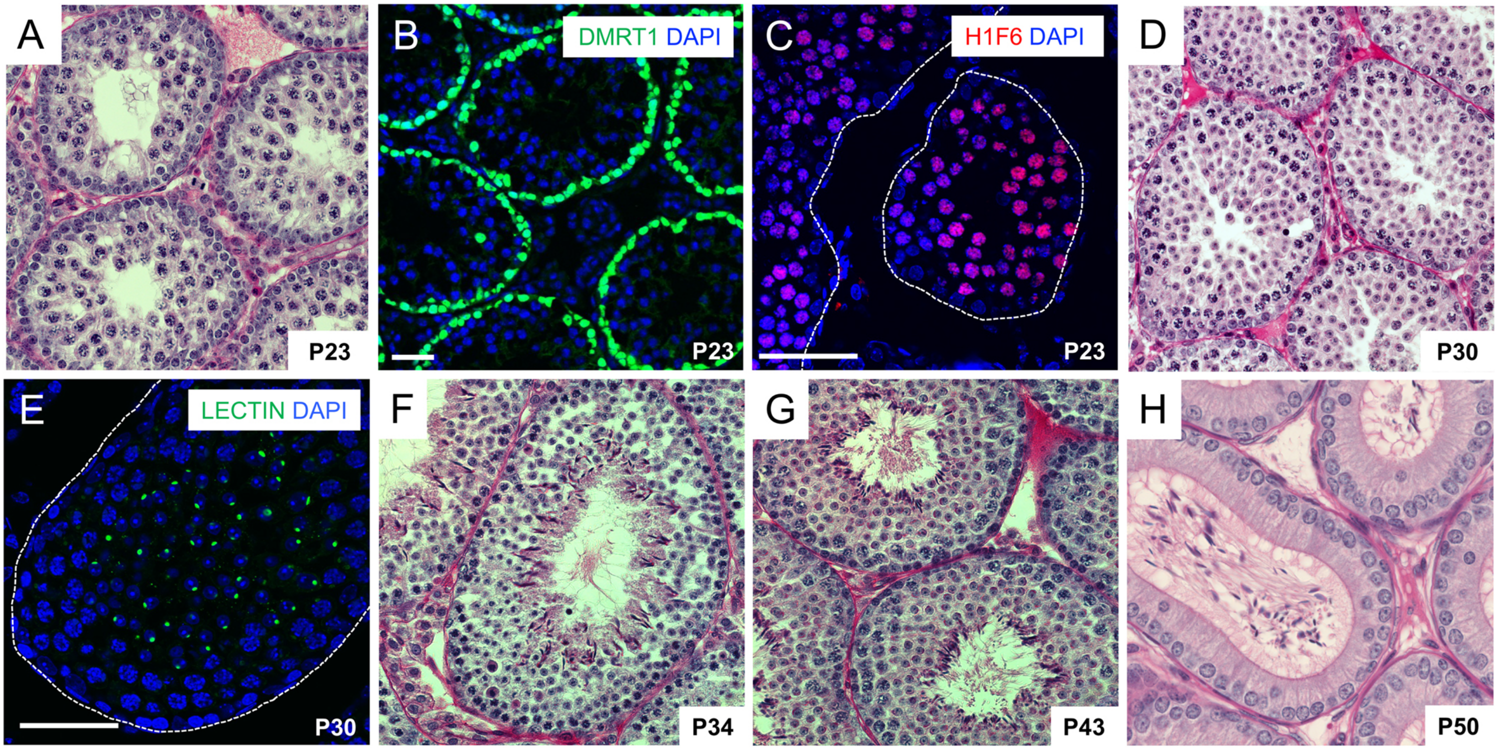

Fig. S1. Timeline for the completion of synchronized spermatogenesis in RA-sufficient mice. Sections from Bouin's-fixed testes (**A, D, F, G**) and epididymis (**H**) were stained with PAS. (**B, C, E**) Immunostaining was done on cryosections, with markers (and corresponding colors) indicated on each image. Nuclei were counterstained with DAPI (blue). Scale bars = 25 µm.

Development • Supplementary information

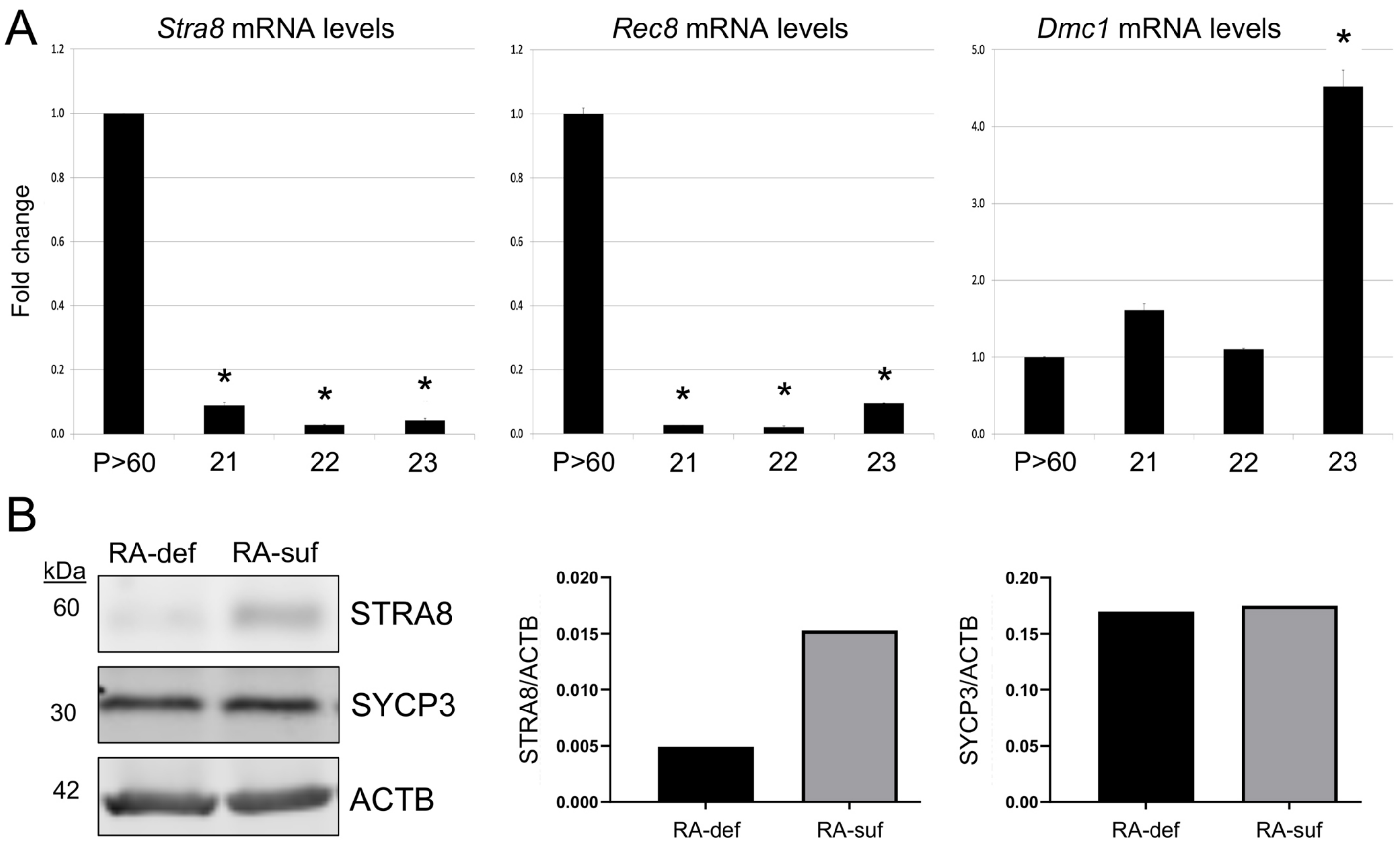

Fig. S2. Confirming testis RA deficiency. (**A**) RNA from RA-deficient testes and WT adult (P>60) testes were used for qRT-PCR to quantify *Stra8*, *Rec8*, and *Dmc1*. (**B**) Immunoblotting was done with whole testis lysates from P19 RA-sufficient and -deficient testes to detect STRA8 and SYCP, and ACTB was used as a loading control. Densitometry results are shown to the right of the immunoblots.

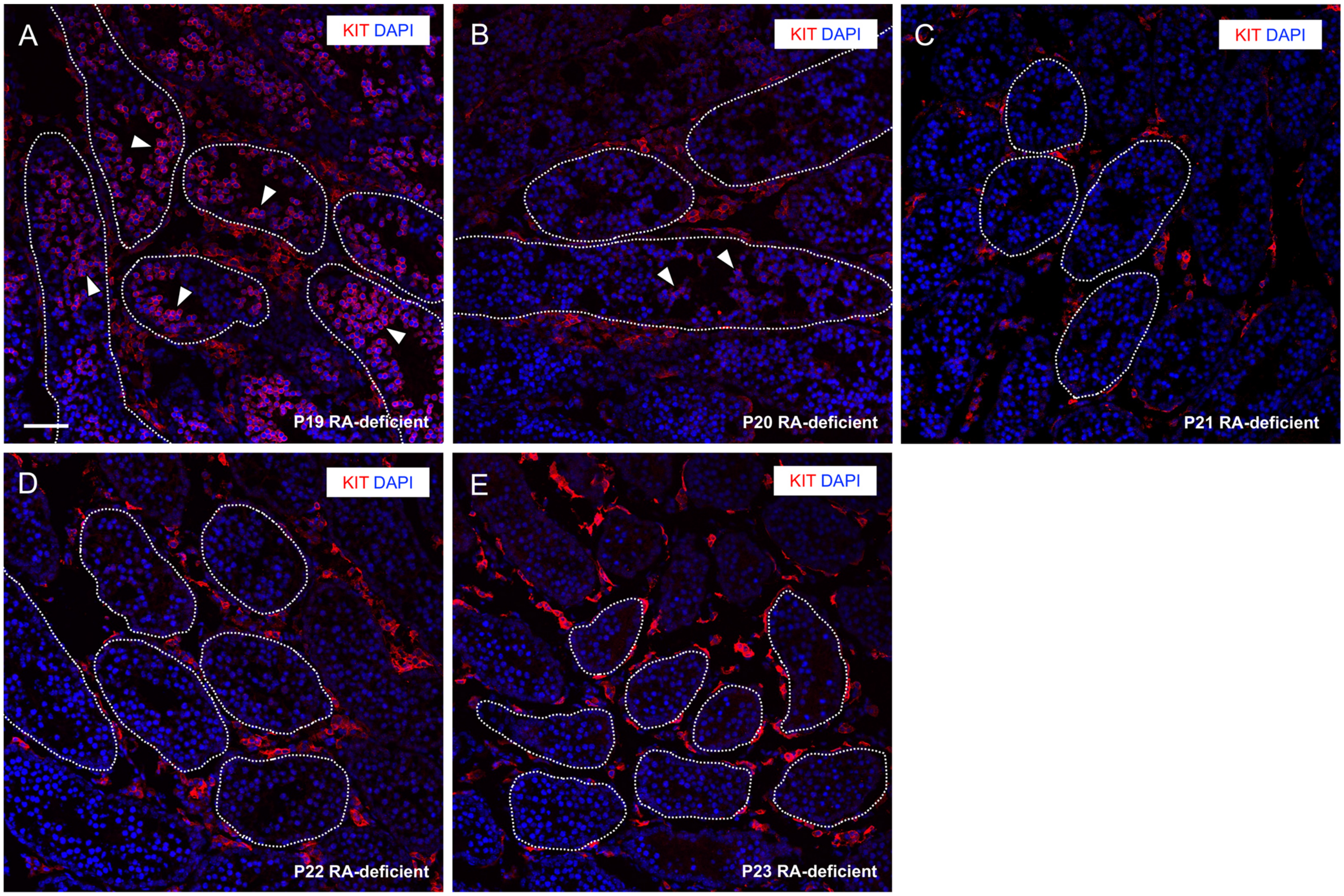

Fig. S3. Confirming lack of RA response after initiation of meiosis. (**A-E**) Testis sections from mice with synchronized RA-deficient spermatogenesis were fixed in PFA, and cryosections immunostained for KIT (red), and nuclei counterstained with DAPI (blue). Ages are indicated on each panel. Representative seminiferous tubules are outlined (white dashed lines). Exemplary spermatocytes with decreasing amounts (over time) of residual KIT are indicated with white arrows in A-B. Scale bars = 50 µm.

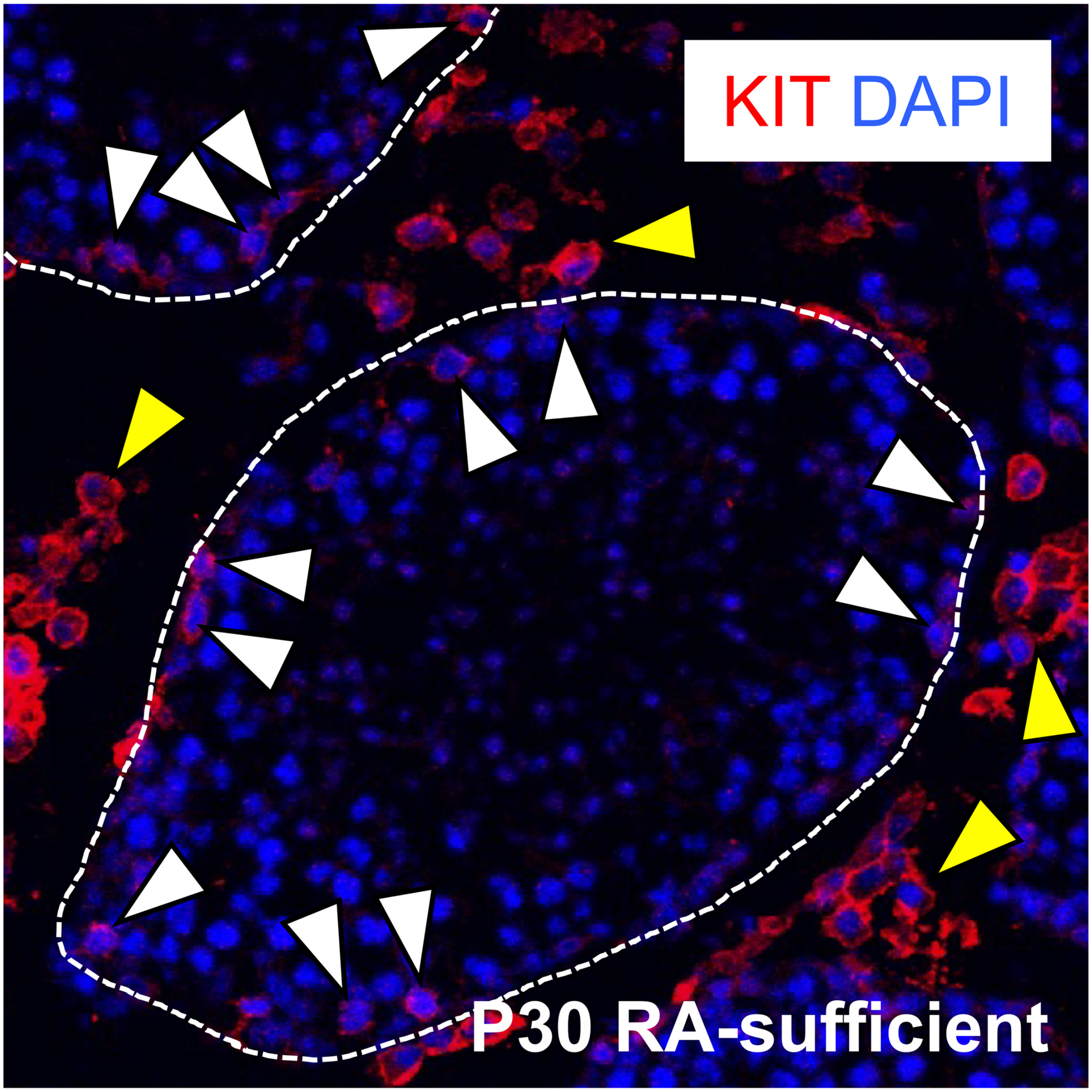

Fig. S4. The next generation of spermatogonia are differentiating KIT+ in P30 RA-sufficient testes. Testis section from mice with synchronized RA-sufficient spermatogenesis were fixed in PFA, and cryosections immunostained for KIT (red), and nuclei counterstained with DAPI (blue). Representative seminiferous tubules are outlined (white dashed lines). Exemplary KIT+ differentiating spermatogonia are indicated with white arrowheads, and typical KIT+ interstitial cells are indicated with yellow arrowheads.

P23 RA-sufficient

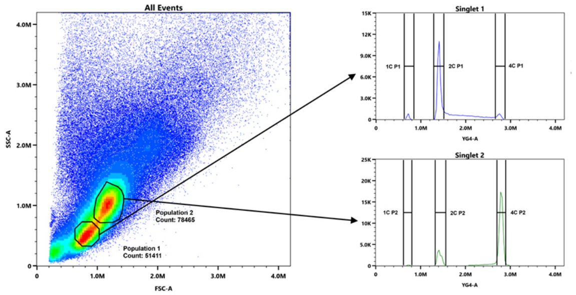

Population 1

DNA Content	Median Value	% CV
1C	735.88	2.92
2C	1431.36	2.25
4C	2777.09	1.89

Population 2

DNA Content	Median Value	% CV
1C	754.36	2.25
2C	1443.10	3.0
4C	2833.24	1.33

P30 RA-sufficient

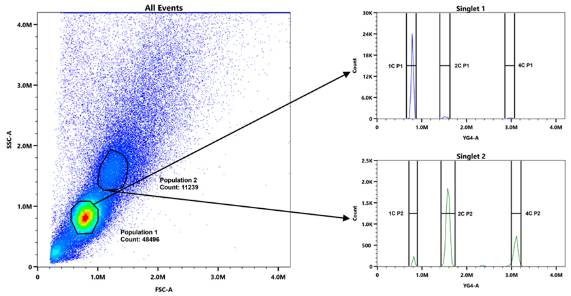

Population 1

DNA Content	Median Value	% CV
1C	812.07	2.50
2C	1552.66	2.57
4C	3015.40	1.45

Population 2

DNA Content	Median Value	% CV
1C	837.84	3.00
2C	1608.10	2.41
4C	3138.24	1.41

P23 RA-deficient

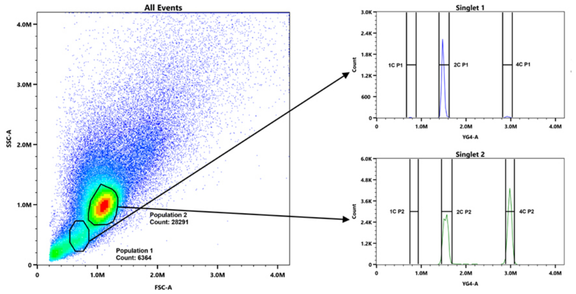

Population 1

DNA Content	Median Value	% CV
1C	766.88	6.94
2C	1509.54	1.84
4C	2958.46	1.34

Population 2

DNA Content	Median Value	% CV
1C	851.59	5.58
2C	1576.03	3.06
4C	3019.24	1.30

P30 RA-deficient

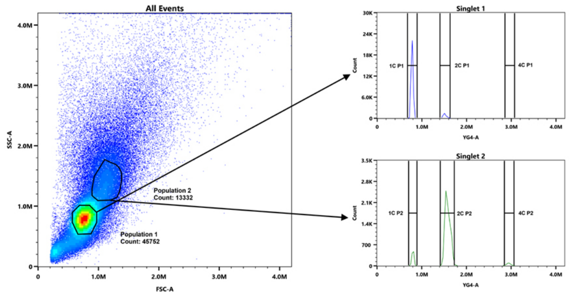

Population 1

DNA Content	Median Value	% CV
1C	803.85	2.45
2C	1535.43	2.32
4C	2976.66	1.58

Population 2

DNA Content	Median Value	% CV
1C	826.92	2.53
2C	1601.64	3.47
4C	2979.65	1.55

Fig. S5. Ploidy determination by flow cytometry. Single cell suspension from P23 and P30 testes with RA-sufficient vs -deficient synchronized spermatogenesis stained with PI. MFI values indicate DNA doubling between 1C and 2C, and 2C and 4C.

Development • Supplementary information

Table S1. (XLS) Merged analyses of pairwise DEG comparisons of A_{undiff} vs A_1 differentiating spermatogonia, A_1 vs A_3 differentiating spermatogonia, A_3 vs In/B differentiating spermatogonia, and In/ B differentiating spermatogonia vs preleptotene spermatocytes.

Click here to download Table S1

Table S2. (XLS) List of genes with differentially expressed mRNAs between A_{undiff} and A_1 differentiating spermatogonia. The first tab lists all DEGs and their corresponding unbiased cluster, while each subsequent tab reflects pathway analysis of the genes contained in the noted cluster.

Click here to download Table S2

Table S3. (XLS) List of genes with differentially expressed mRNAs between A_1 and A_3 differentiating spermatogonia. The first tab lists all DEGs and their corresponding unbiased cluster, while the second tab reflects pathway analysis output for all six clusters.

Click here to download Table S3

Table S4. (XLS) List of genes with differentially expressed mRNAs between A_3 and In/B differentiating spermatogonia. The first tab lists all DEGs and their corresponding unbiased cluster, while each subsequent tab reflects pathway analysis of the genes contained in the noted cluster.

Click here to download Table S4

Table S5. (XLS) List of genes with differentially expressed mRNAs between In/B differentiating spermatogonia and preleptotene spermatocytes. The first tab lists all DEGs and their corresponding unbiased cluster, while each subsequent tab reflects pathway analysis of the genes contained in the noted cluster.

Click here to download Table S5

Development • Supplementary information

Table S6. (XLS) DESeq2 comparisons of differential gene expression between In/B differentiating spermatogonia vs RA-sufficient preleptotene spermatocytes and RA-deficient preleptotene spermatocytes.

Click here to download Table S6

Table S7. (XLS) List of genes with differentially expressed mRNAs between In/B differentiating spermatogonia and preleptotene spermatocytes but were unchanged in RA-sufficient vs -deficient testes. The first tab lists all DEGs and their corresponding unbiased cluster, while each subsequent tab reflects pathway analysis of the genes contained in the noted cluster.

Click here to download Table S7

Table S8. (XLS) List of genes with differentially expressed mRNAs in RA-sufficient preleptotene spermatocytes vs In/B differentiating spermatogonia but were not significantly changed in RA-deficient preleptotene spermatocytes. The first tab lists all DEGs and their corresponding unbiased cluster, while each subsequent tab reflects pathway analysis of the genes contained in the noted cluster.

Click here to download Table S8
